# Supplementary material for: Synaptopodin-2 Isoforms Have Specific Binding Partners and Display Distinct, Muscle Cell Type-Specific Expression Patterns
Source: Cells. 2023 Dec 30;13(1):85. doi: 10.3390/cells13010085 (PMC10778272; doi:10.3390/cells13010085)
Supplement: Supplementary file 1 [file cells-13-00085-s001.zip › cells-2756951-supplementary.pdf]

Supplementary materials

Synaptopodin-2 Isoforms Have Specific Binding Partners and Display Distinct, Muscle Cell Type-Specific Expression Patterns

Keerthika Lohanadan, Marvin Assent, Anja Linnemann, Julia Schuld, Lukas C. Heukamp, Karsten Krause, Matthias Vorgerd, Jens Reimann, Anne Schä nzer,Gregor Kirfel, Dieter O. Fürst and Peter F.M. van der Ven

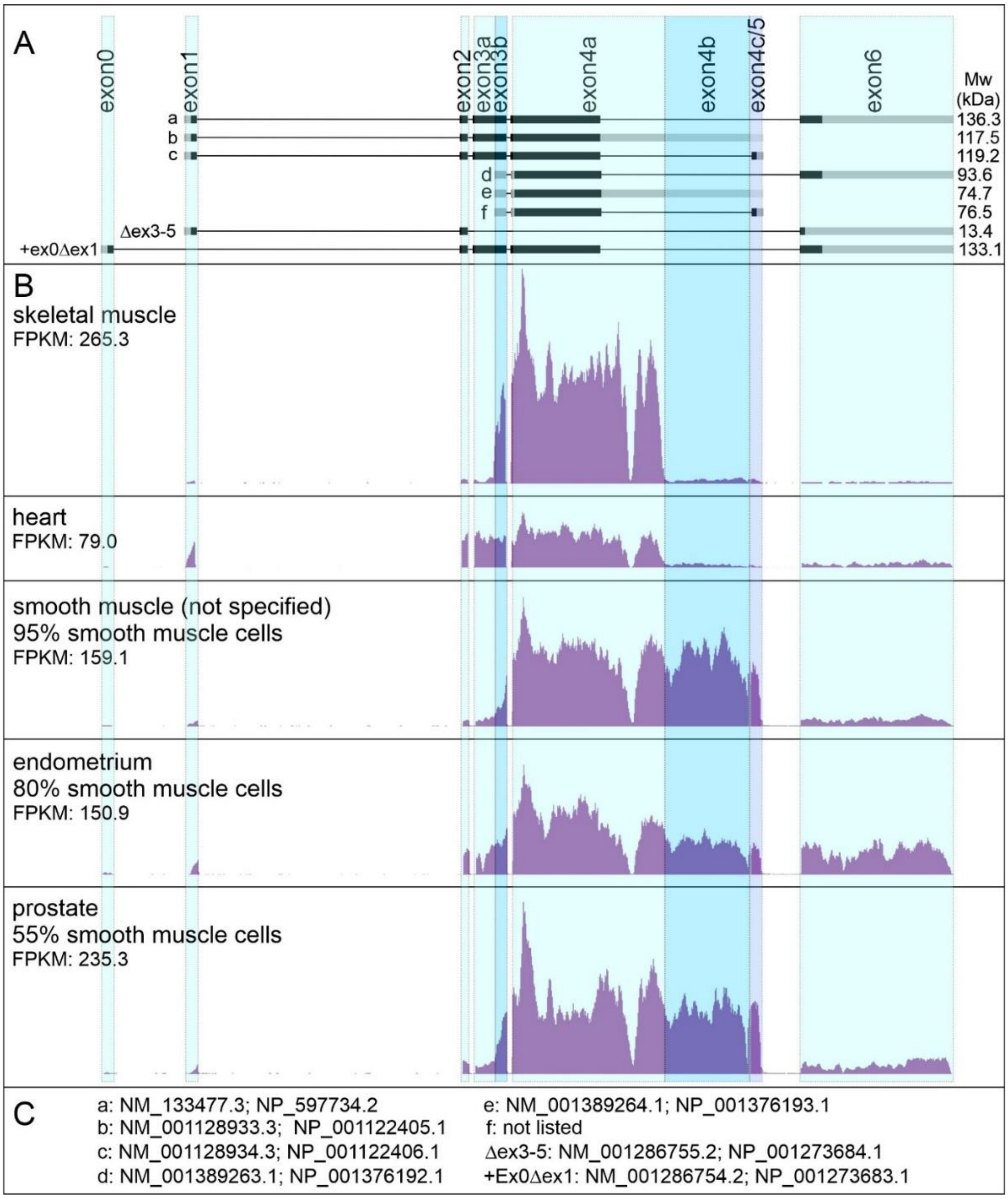

Figure S1. Expression of SYNPO2 isoforms according to HPA v15. (A) *Structure of the SYNPO2 gene and a summary of (predicted) isoforms. At the right the calculated molecular mass of the individual isoforms is given.* ***(B)*** *Representative expression patterns of the individual exons in different muscle cell-containing tissues as presented by the Human Protein Atlas v15. The putative exon 0, that is localized upstream of exon 1 is expressed at hardly detectable levels in all tissues, but not in skeletal muscle. Only in the heart, exons 1, 2 and 3 encoding the PDZ domain are highly expressed, at levels comparable to exon 4. The expression in skeletal muscle is very low, while smooth muscle-containing tissues express low to medium levels. Exon 4a is part of all isoforms. Exon 4b, that extends the 3'UTR of SYNPO2 mRNA, without changing the open reading frame, appears to be mainly expressed in smooth muscle cell-containing tissues. These data do not provide any evidence for expression of the isoform in which exon 4a is spliced to exon 5. Instead, exon 5 might be incorporated in the extended 3' UTR. Exon 6 is mainly expressed in smooth muscle cells. In the endometrium its expression level is comparable to that of exon 4, whereas in smooth muscle cells of the prostate exon 6 is hardly expressed. Graphs were made to scale. The actual expression of SYNPO2 in FKPM, and the percentage of smooth muscle cells in the analyzed specimen is given.* ***(C)*** *RefSeq accession numbers of the individual mRNA (NM) and protein (NP) isoforms as listed by the NCBI.*

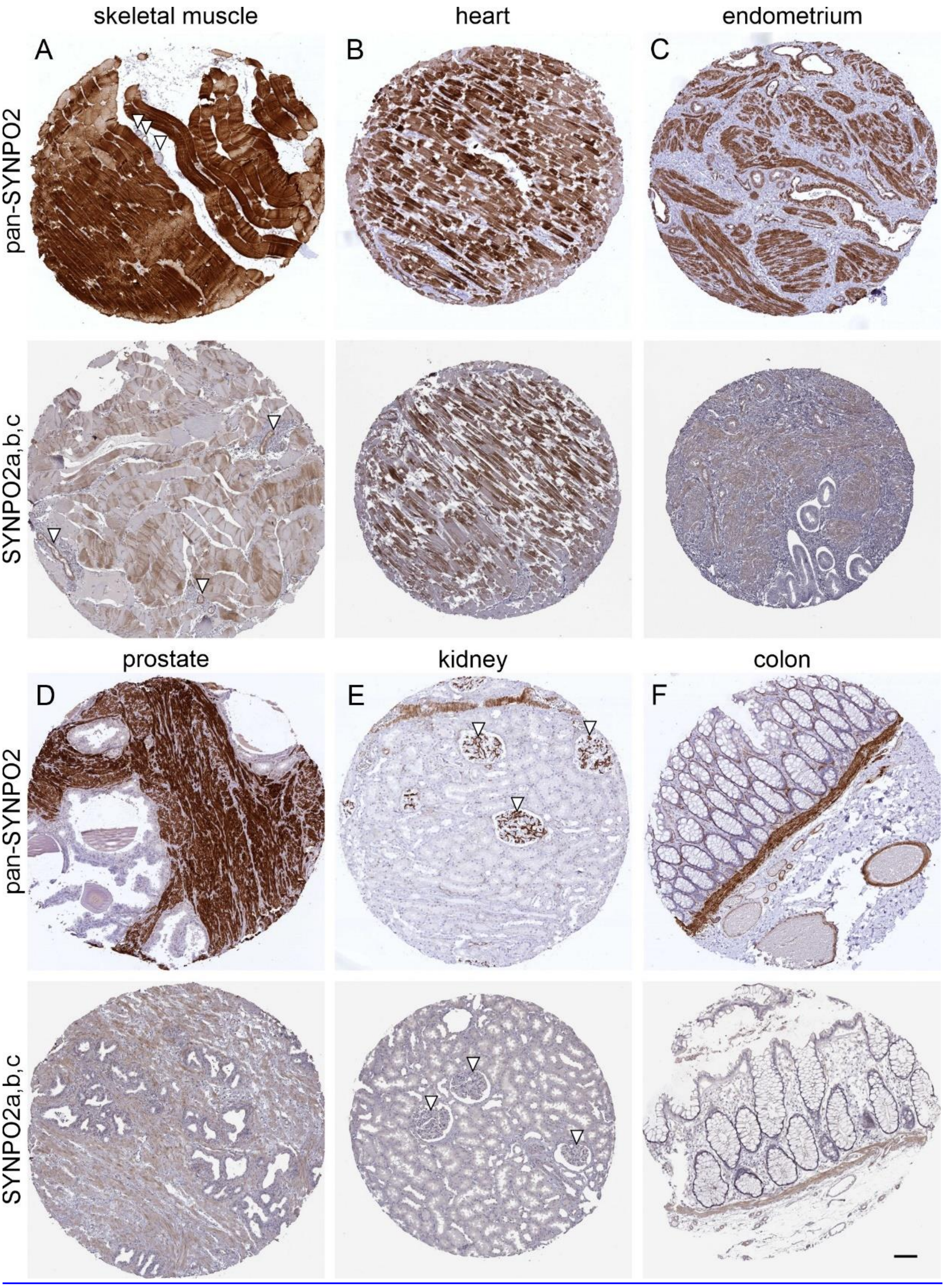

Figure S2. Differential expression of SYNPO2 isoforms with and without PDZ domain in different tissues according to HPA (v23). *Shown are sections prepared from (A) skeletal muscle, (B) heart muscle (C) smooth muscle, (D) prostate, (E) kidney and (F) colon either stained with an antibody that recognizes all SYNPO2 isoforms (HPA030665, pan-SYNPO2; upper panels) or an antibody specific for the PDZ-containing isoforms SYNPO2a,b,c (HPA068563, lower panels). The only tissue showing comparable staining with both antibodies is the heart (B), which is in agreement with RNA expression data. In all other tissues (A,C,D,E,F), cells stained by pan-SYNPO2 are not, or only weakly stained by anti-SYNPO2a,b,c. Note expression of SYNPO2a,b,c in blood vessels (arrowheads in A) and complete absence of these variants in glomeruli in the kidney (arrowheads in E). Bar: 100 μm*

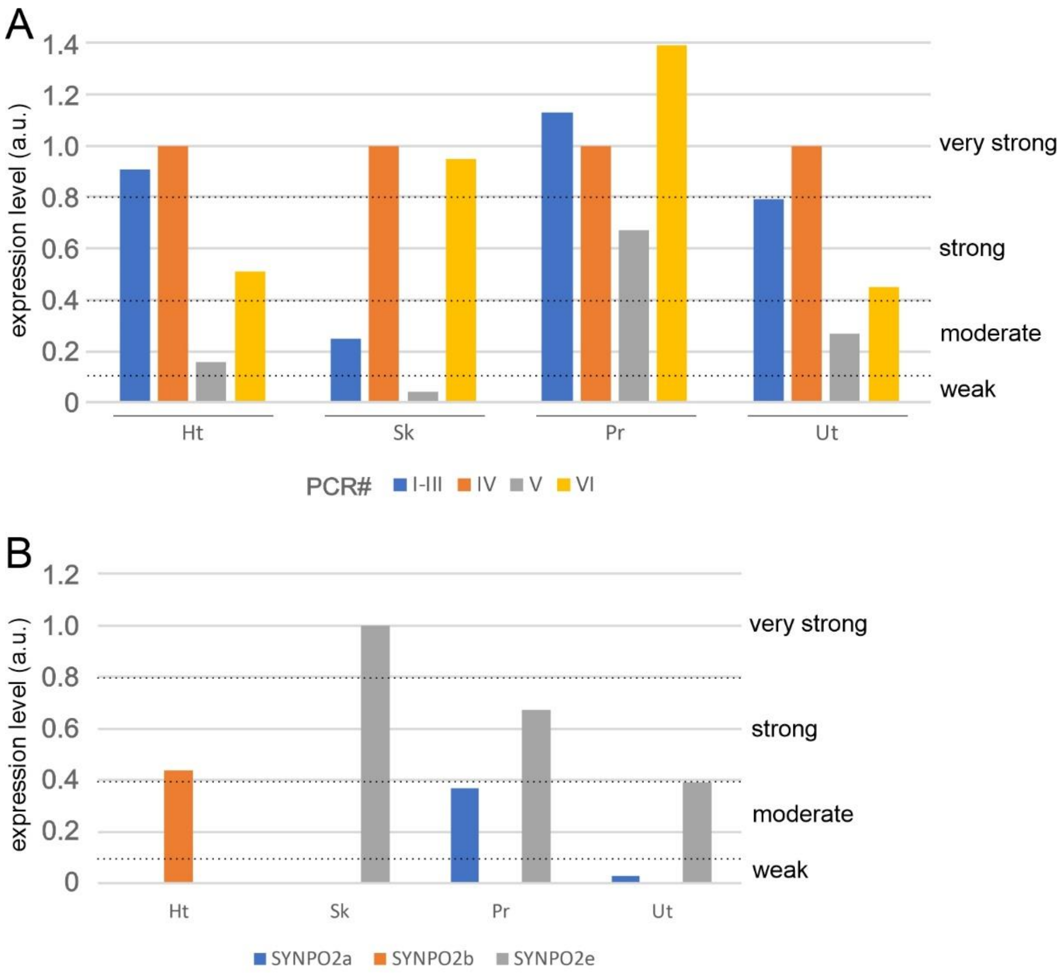

Figure S3. Densitometry of RT-PCR and western blotting experiments. *(A) Densitometric analysis of representative agarose gels obtained from the RT-PCR experiments. Bands corresponding to PCR IV (all isoforms) were normalized to 1.0. The quantity of all other bands was divided by the signal of PCR IV to establish the relative quantity of each band and isoform. For PCRs I-III, representing the isoforms containing the PDZ domain (a-c), values were averaged. Relative expression levels were categorized as indicated. (B) Densitometric analysis of representative western blots. To estimate the relative abundance of various SYNPO2 isoforms in different tissues at the protein level, representative western blots stained with an antibody recognizing all SYNPO2 isoforms, were analyzed by densitometry. The highest protein level (i.e. that in skeletal muscle) was set to 100%, and the relative levels of SYNPO2 isoforms in other tissues were calculated. Relative expression levels were classified as indicated. a.u.: arbitrary units*

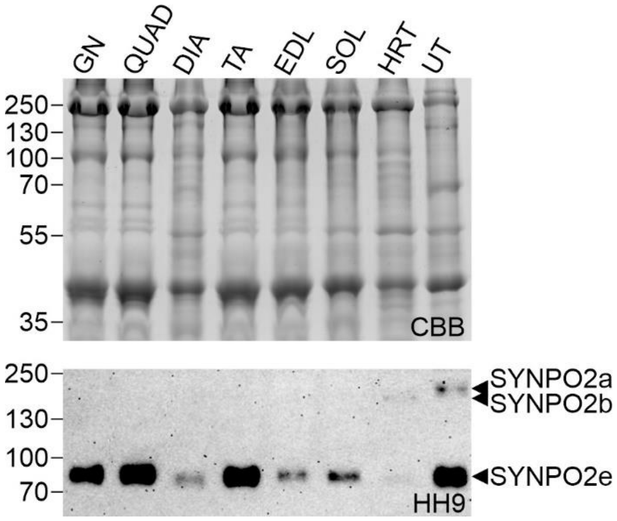

Figure S4. Expression of SYNPO2 varies considerably in different mouse tissues. *The upper panel shows a polyacrylamide gel stained with Coomassie Brilliant Blue stained gel. the lower panel an identical gel blotted to nitrocellulose and stained with HH9, our pan-SYNPO2 monoclonal mouse antibody. Note highly different SYNPO2 levels in the different skeletal muscles. The mouse heart (HRT) expresses low levels of two isoforms (SYNPO2e and SYNPO2b), while mouse uterus (UT) mainly expresses SYNPO2e, and in addition medium levels of SYNPO2a. GN: gastrocnemius, QUAD: quadriceps, DIA: diaphragm, TA: tibialis anterior, EDL: extensor digitorum longus, SOL: soleus, HRT: heart, UT: uterus*

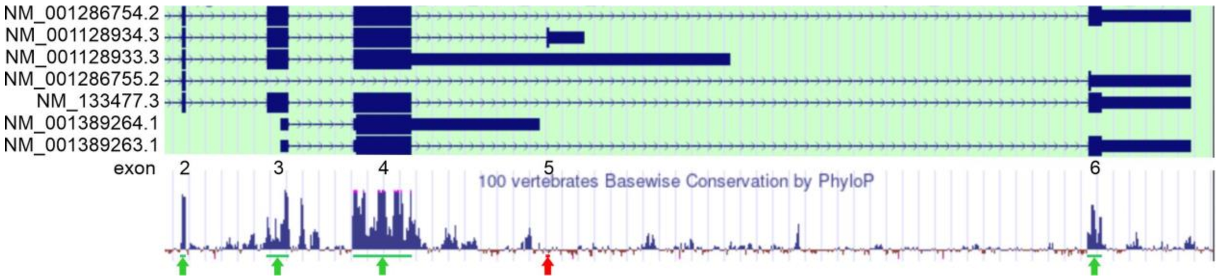

Figure S5. Conservation of SYNPO2 exon sequences in vertebrates. *Shown is the organization of the SYNPO2 gene between exon 2 and exon6, and the exon conservation in 100 vertebrates as analyzed by PhyloP. Note high conservation of exons 2, 3, and the protein encoding part of exons 4 and 6 (green arrows) and very limited conservation of exon 5 (red arrow).*
